# Editorial: Ascorbate metabolism in plants

**DOI:** 10.3389/fpls.2023.1208209

**Published:** 2023-05-26

**Authors:** Luis Miguel Morales Mazorra, Mara Menezes de Assis Gomes, José Hélio Costa, Ricardo Antônio Ayub, Jurandi Gonçalves de Oliveira

**Affiliations:** ^1^ Plant Physiology Institute, National University of La Plata, La Plata, Argentina; ^2^ Laboratório de Melhoramento Genético Vegetal, Centro de Ciências e Tecnologias Agropecuárias, Universidade Estadual do Norte Fluminense Darcy Ribeiro (UENF), Rio de Janeiro, Brazil; ^3^ Functional Genomics and Bioinformatics, Department of Biochemistry and Molecular Biology, Federal University of Ceará, Fortaleza, Ceará, Brazil; ^4^ Non-Institutional Competence Focus (NICFocus) ‘Functional Cell Reprogramming and Organism Plasticity’ (FunCROP), coordinated from Foros de Vale de Figueira, Alentejo, Portugal; ^5^ Laboratório de Biotecnologia Aplicada a Fruticultura, Departamento de Fitotecnia e Fitossanidade, Universidade Estadual de Ponta Grossa, Ponta Grossa, Paraná, Brazil

**Keywords:** ascorbic acid synthesis, ascorbic acid production, GDP-D-mannose pyrophosphorylase, increasing ascorbic acid content in plants, l-galactono-1,4-lactone dehydrogenase

Ascorbic acid (AsA), commonly called vitamin C, is an important antioxidant essential to animal and plant metabolism. Humans have lost the ability for AsA synthesis so this molecule should be supplied by diet. A lot of synthetic methods have been proposed to produce AsA. Nevertheless, they present some limitations and plants currently are the main source of vitamin C for humans. The main approaches examined to increase the content of AsA in plant tissues include the increase of “*de novo*” AsA synthesis, the improvement of the AsA recycling and the decrease of the AsA degradation.

In this special issue, a total of two research articles and three reviews are brought together to add new knowledge to our understanding about the ascorbate metabolism in plants. The research works were focused on aspects of the AsA biosynthesis in different plant species whereas reviews presented elaborated compilations and new perspectives of the ongoing progress in this area of research.

The pathways of AsA synthesis have not been fully elucidated in plants. Indeed, four possible pathways have been proposed, with the so-called “Smirnoff-Wheeler” or “d-mannose/l-galactose” pathway being the best characterized. Particularly, the plant GDP-d-mannose pyrophosphorylase (GMPase) catalyzes a committed step in AsA biosynthesis. The research work presented by Zhang et al. revealed previously uncharacterized features of crystal structures of the Arabidopsis GMPase, providing structural information that may help to understand the different mechanisms involved in the regulation of this enzyme.

On the other hand, Lu et al. have described original research on jujube (*Ziziphus jujuba* Mill.) fruits, in which transcriptome and metabolite analyses were performed to detect changes in genes and metabolites during jujube fruit development. They found genes associated with AsA synthesis and recycling and identified three AsA-related gene networks/module co-expression patterns. The work looked very promising considering that the role of these genes has not been reported before in this fruit plant.

The poor understanding of the tight regulation of AsA synthesis in plants is considered a main factor that limits the strategies to increase AsA content by modifying AsA synthesis. In fact, the success of increasing AsA content by modifying AsA synthesis in plants has been limited. Castro et al. focused the review on the genetic and biochemical strategies used by plant cells for regulating AsA biosynthesis through the l-galactose pathway. They summarized the main pathway of AsA synthesis, highlighting the key enzymes and metabolites. In addition, this review summarizes various aspects of the regulation of AsA synthesis, including transcriptional and post-transcriptional regulation, protein function, enzymatic activity, subcellular distribution, and other related factors.

Terzaghi and Tullio have offered an interesting perspective about the many attempts made so far to improve AsA production/content in plants. They covered published studies related to the manipulation of AsA content by altering both the main and alternative AsA synthesis pathways. Their review has discussed examples of over-expressions of biosynthetic-genes or AsA recycling genes, which were mostly not effective to increase AsA in an amount that could meet the needs of human nutrition. Also, the main pathways of AsA synthesis as well as key regulation factors were summarized. They raise the question of why unexpected consequences can be observed when a pure biotechnological approach is used without taking into account the specific features of the AsA system in plants. In particular, the fact of that plant performance is altered as result of high AsA level, probably due to AsA-dependent feed-back regulation.

The final step of AsA synthesis occurs associated with the mitochondrial electron transport chain. This step is catalyzed by the l-galactono-1,4-lactone dehydrogenase (l-GalLDH), an enzyme that can introduce electrons directly into the respiratory chain. The possible consequences of the link between AsA synthesis and mitochondrial activity in terms of plant performance have not received much attention. Matos et al. offered a view about the possible effects of this link between AsA synthesis and the mitochondrial respiration. They discussed examples where metabolic alterations of the respiratory activity and the capacity of AsA synthesis seemed to be connected.

## Concluding remarks

The research articles and reviews in this Research Topic mainly focused on the control of AsA production in plants through synthesis and recycling, with relatively less attention given to other aspects of plant metabolism. As suggested in the three reviews, the tight regulation of AsA biosynthesis poses a challenger for increasing AsA content in plants. Possible factors, such as feedback inhibition by AsA or regulation by the mitochondrial electron flux, require further investigation. In conclusion, this Research Topic sheds light on the complex regulation, biosynthesis, and plant production of AsA, emphasizing the need for continued exploration of this important area of research.

## Author contributions

LMMM and JGO planned the editorial. LMMM wrote and JGO edited the editorial. All authors contributed to the editorial and approved the submitted version.

